# New insights into the role of dietary triglyceride absorption in obesity and metabolic diseases

**DOI:** 10.3389/fphar.2023.1097835

**Published:** 2023-02-02

**Authors:** Xiaojing Li, Qiaohong Liu, Yuqing Pan, Si Chen, Yu Zhao, Yiyang Hu

**Affiliations:** ^1^ Key Laboratory of Liver and Kidney Diseases (Ministry of Education), Institute of Liver Diseases, Shanghai Key Laboratory of Traditional Chinese Clinical Medicine, Shuguang Hospital Affiliated to Shanghai University of Traditional Chinese Medicine, Shanghai, China; ^2^ Institute of Clinical Pharmacology, Shuguang Hospital Affiliated to Shanghai University of Traditional Chinese Medicine, Shanghai, China

**Keywords:** chylomicron, dietary fat absorption, enterocyte, obesity, triglycerides

## Abstract

The incidence of obesity and associated metabolic diseases is increasing globally, adversely affecting human health. Dietary fats, especially triglycerides, are an important source of energy for the body, and the intestine absorbs lipids through a series of orderly and complex steps. A long-term high-fat diet leads to intestinal dysfunction, inducing obesity and metabolic disorders. Therefore, regulating dietary triglycerides absorption is a promising therapeutic strategy. In this review, we will discuss diverse aspects of the dietary triglycerides hydrolysis, fatty acid uptake, triglycerides resynthesis, chylomicron assembly, trafficking, and secretion processes in intestinal epithelial cells, as well as potential targets in this process that may influence dietary fat-induced obesity and metabolic diseases. We also mention the possible shortcomings and deficiencies in modulating dietary lipid absorption targets to provide a better understanding of their administrability as drugs in obesity and related metabolic disorders.

## 1 Introduction

Obesity is a growing global health crisis. Obesity leads to a number of comorbidities such as non-alcoholic fatty liver disease (NAFLD), diabetes, hyperlipidemia, cardiovascular disease, and cancer, causing an enormous health burden and harm life quality (Chooi et al., 2019). To curb the obesity epidemic, lifestyle intervention becomes a lifelong process. However, obesity is difficult to control and remains a serious public health problem worldwide (Seidell and Halberstadt, 2015).

Increased food energy consumption is the leading cause of obesity and metabolic diseases (Vandevijvere et al., 2015). Triglyceride (TG) is the main dietary lipid, and nearly 90%–95% of the energy produced by fat is derived from TG (Iqbal and Hussain, 2009). Clinical and animal studies have demonstrated the importance of a high-fat diet (HFD) in developing obesity and metabolic disorders. In animal studies, HFD has been widely used to induce obesity, NAFLD, type 2 diabetes mellitus, hyperinsulinemia, and hyperlipidemia in mouse models (Heydemann, 2016; Recena Aydos et al., 2019; Li et al., 2020). Increased fat absorption is one of the primary impetuses of obesity and metabolic diseases (Luo et al., 2018). In contrast, restricting TG absorption improves the obesity phenotype.

The intestine, which absorbs nutrients, is of principal importance in regulating dietary fat absorption. Nearly 95% of dietary TG can be absorbed and undergo digestion, uptake, resynthesis, and secretion into the circulation in chylomicron (CM) (Mansbach and Siddiqi, 2016; Zembroski et al., 2021). Dietary TG is essential for the maintenance of systemic lipid homeostasis. Excessive intake of TG causes systemic metabolic disorders and intestinal lipid metabolism disorders. In humans, as little as 3 days of HFD can significantly decrease gastrointestinal transit time (Clegg et al., 2011). Moreover, a chronic HFD markedly affects intestinal physiology and even influences the absorption of other macronutrients. The regulation of essential genes involved in this process may disrupt the entry of dietary TG into the circulatory system. In this review, we have insight into the role of dietary TG absorption in obesity and metabolic diseases. We will overview the dietary TG absorption process in the intestine, especially focusing on the interaction between TG absorption, intestinal function, and metabolic diseases, as well as the key proteins that regulate intestinal lipid mobilization and metabolism. We hope to provide a novel perspective for the preventing and treating of obesity and metabolic diseases.

## 2 Overview of dietary triglyceride absorption process in intestine

### 2.1 Dietary triglyceride digestion

**FIGURE 1:**
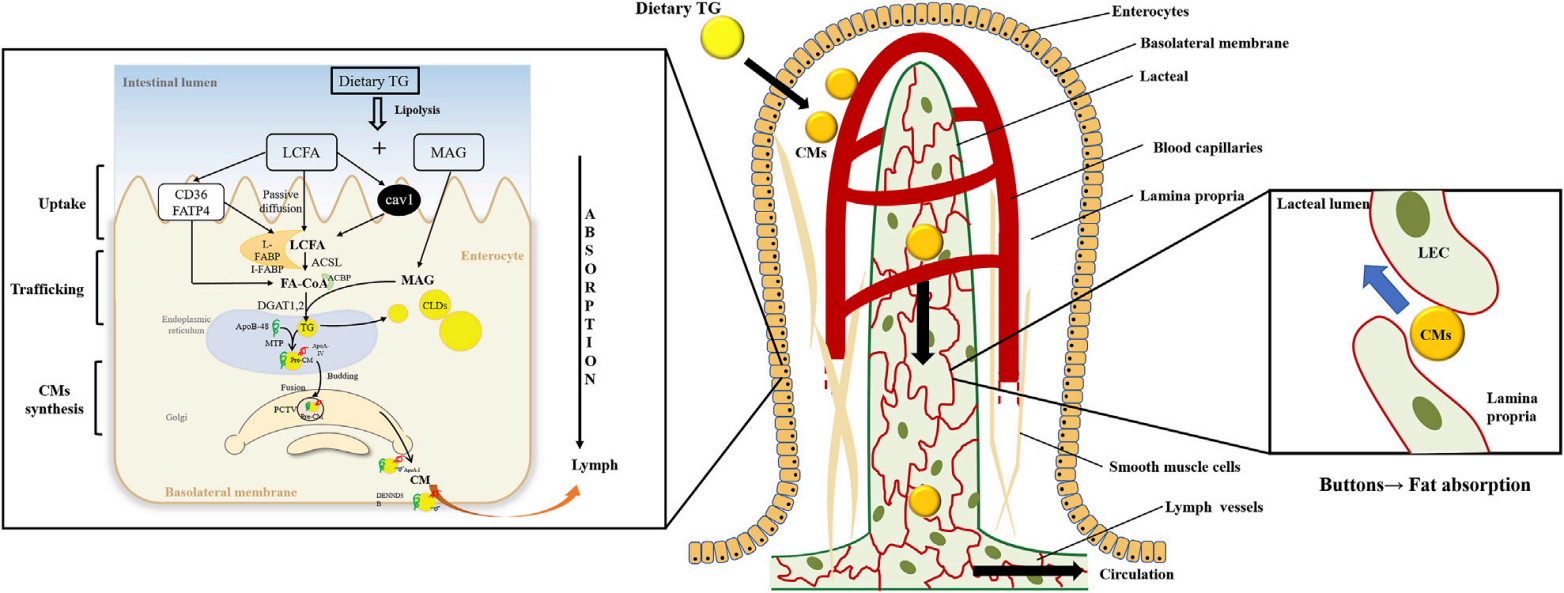
Dietary triglyceride transport from the intestinal lumen, enterocytes, and lacteals. Dietary triglyceride (TG) is hydrolyzed into long-chain fatty acids (LCFAs) and monoacylglycerols (MAGs) in the intestinal lumen. The enterocytes uptake the digestion products and traffic them into the endoplasmic reticulum (ER). And TG is resynthesized in the ER, which may bud off the ER membrane to form cytoplasmic lipid droplets (CLDs) or be assembled to form pre-chylomicrons (pre-CMs). The pre-CMs are transported to Golgi in pre-chylomicron transport vesicle (PCTV) for further mature. Then mature chylomicrons (CMs) exit the basolateral membrane to lamina propria. CMs are transported into the lacteals through the buttons junction of lymphatic endothelial cells (LECs) and secreted into the lymphatic vessels before entering circulations.

TG digestion begins upon contacting the lingual lipase secreted by the lingual gland. Digestion continues under the action of gastric enzymes in the stomach. The coarse fat emulsion enters the duodenum as lipid droplets and is solubilized by the action of bile acid. TG is further digested and hydrolyzed in the lumen by several pancreatic lipases (PL) to yield *sn*-2-monoacylglycerol (2-MAG) and fatty acids (FAs). 2-MAG and long-chain FA (LCFA) are taken up by intestinal epithelial cells effectively (Figure 1).

### 2.2 Long-chain fatty acid uptake and traffic

Transport of LCFA and MAG in enterocytes occurs by passive diffusion. In addition, transporters promote LCFA take-up, especially when the concentrations of LCFA are low in the lumen. Various LCFA transporters, including the cluster of differentiation 36 (CD36), fatty acid transport protein 4 (FATP4), and the plasma membrane-associated fatty acid-binding protein, are involved in the transportation process (Stahl et al., 1999; Drover et al., 2005). Moreover, Caveolae-mediated endocytosis also contributes to FAs uptake, and caveolin-1 plays a vital role in forming caveolae (Parton and del Pozo, 2013). Although specific 2-MAG transporter has not been reported, some *in vitro* experiments suggest that FAs transporters are partially involved in this process (Murota et al., 2001; Murota K, 2005).

LCFAs and 2-MAG traverse across the cytoplasm to reach the endoplasmic reticulum (ER) membrane in enterocytes. Cytoplasmic fatty acid-binding proteins such as intestinal FABP (I-FABP) and liver FABP (L-FABP) are involved in intracellular LCFAs transportation (Storch and Corsico, 2008).

### 2.3 Intracellular triglyceride resynthesis

The first step of TG resynthesis is adding the coenzyme A (CoA) group to the LCFAs, which is catalyzed by long-chain acyl-CoA synthetases (ACSL) (Ellis et al., 2010). FATP4 has acyl CoA synthetase activity and is also involved in FAs acylation (Milger et al., 2006). The newly generated FA-CoA is bound to an acyl-CoA binding protein, and this process can regulate cellular FA-CoA disposal (Niot et al., 2009).

In the ER membrane, two multi-enzymatic systems catalyze FA-CoA re-esterified into TG: the glycerol-3-phosphate pathway and the monoacylglycerol pathway. In enterocytes, TG resynthesis is mainly along the monoacylglycerol pathway, and nearly 80% of the resynthesized TG is produced from this pathway (Kern and Borgstrom, 1965). Monoacylglycerol acyltransferase (MGAT) is a key enzyme that transports FA-acyl-CoA to 2-MAG to generate DAG. DAG is further esterified by diacylglycerol acyltransferase (DGAT) with an FA-CoA to yield TG (Cases et al., 1998).

### 2.4 Enterocyte cytoplasmic lipid droplets storage

The re-esterified TG in intestinal epithelial cells is either transported to the ER to synthesize lipoproteins or stored in cytoplasmic lipid droplets (CLDs) in a highly dynamic state (Demignot et al., 2014). In enterocytes, the biogenesis mechanisms of CLD remains unclear. This process may consist of re-esterified TG accumulation and lens formation in the ER bilayer, and then the CLD enters the cytoplasm by budding (Zembroski et al., 2021). Intestinal CLDs are divided into different dynamic lipid pools due to three different origin sources: 1) dietary lipids taken up on the apical side of enterocytes; 2) circulation lipids absorbed at the basolateral side of enterocytes; 3) *de novo* FAs synthesis. CLDs synthesized from different sources may undergo different metabolic fate. FAs delivered from the apical side are preferably used for TG synthesis, while the FAs delivered from the basolateral side are used for phospholipid synthesis and preferential oxidation (Storch et al., 2008).

### 2.5 Chylomicron assembly, trafficking, and secretion

The resynthesized TG is transported to the ER to synthesize CMs. In the inner leaflet of ER, microsomal triglyceride transfer protein (MTP) facilitates lipidation of apolipoprotein B-48 (ApoB-48) to form the pre-CM. The pre-CMs is then secreted from the ER and transported to the Golgi *via* pre-CM transport vesicles (PCTVs) for further maturation. In Golgi, pre-CM is further modified by addition of ApoA-I and glycosylating ApoB-48 to form mature CM [reviewed in (D'Aquila et al., 2016)]. Mature CM is excreted from the Golgi in large vesicles and then releases from the basolateral side of the intestinal epithelial cells into the lamina propria by exocytosis. Lacteal drains CMs into the lymphatic system and finally enters into circulation (D'Aquila et al., 2016).

## 3 Impacts of dietary triglyceride on obesity and metabolic diseases

### 3.1 Influence of triglyceride consumption

The amount of dietary TG intake is a decisive factor in obesity and metabolic diseases. The human body has a good absorption and clearance capacity of dietary TG. A single intake of 15 g of dietary TG did not affect postprandial lipids and lipoprotein in healthy adults (Dubois et al., 1998). Although postprandial hyperlipemia is a normal physiological response to acute dietary TG intake, increased TG intake may break the balance, leading to stepwise severe lipidaemia (Dubois et al., 1998). An intake of over 40 g of fat per meal in healthy adults can lead to lipidaemia, which can be exacerbated by successive lipid-containing meals (Dubois et al., 1998). Another study in obese individuals also confirmed that ingesting 40 g of fat can increase the postprandial plasma TG concentration (Vors et al., 2015). Postprandial states are repeated during waking hours, and pathological postprandial hyperlipidemia persists for a long time, which leads to the accumulation of postprandial TG-rich lipoproteins. Their remnants in the circulation may have a profound effect on cardiovascular disease, obesity, and metabolic diseases. In patients with insulin resistance (IR), elevated serum TG is often found in non-fasting state (Moreno-Perez et al., 2021).

### 3.2 Impacts of the composition of fatty acids

**FIGURE 2:**
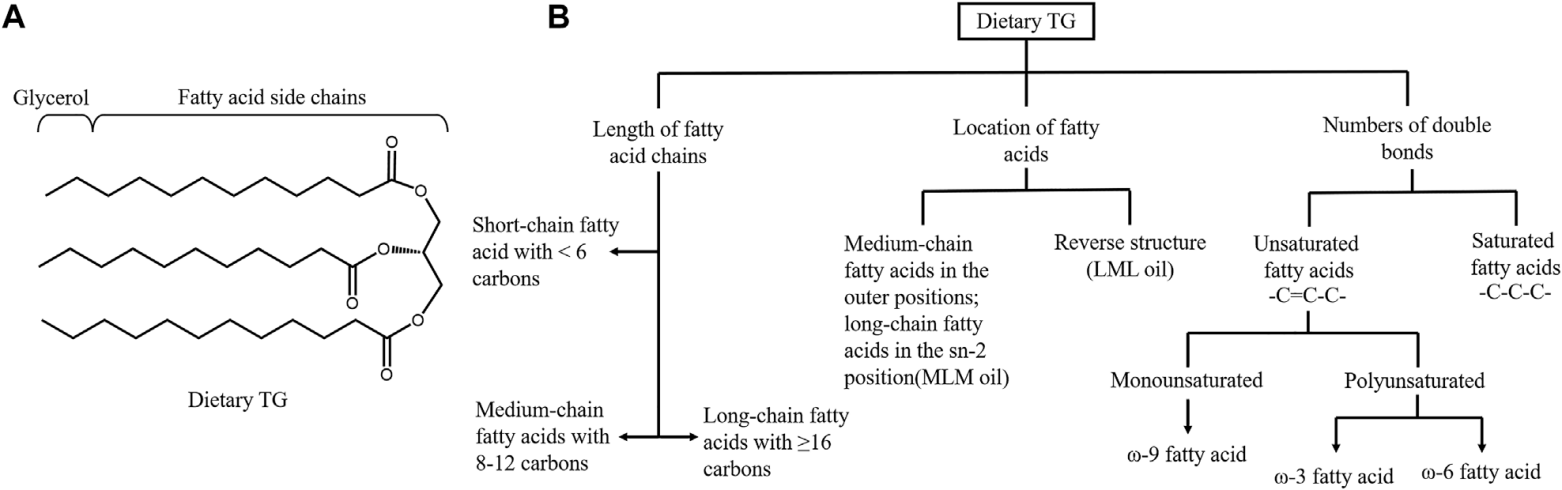
Structure and classification of dietary triglyceride. **(A)** Structure of triglyceride (TG). Pancreatic lipase hydrolyzes the sn-1 and sn-3 positions of TGs to generate sn-2 MGs and free fatty acids. **(B)** Dietary triglyceride can be classified based on the length of fatty acid (FA) chains, the location of different length of FAs in TG and the numbers of double bonds in the hydrocarbon chains. Basing on the numbers of carbons, FAs can be subcategorized into short, medium and long-chain FAs. Three FAs in the TG contribute to the different structured oil, including medium-chain FA in the outer positions of the TG and long-chain FA in the sn-2 position and the reverse structure. Unsaturated FAs contains double bonds while saturated FAs only have single bonds. Unsaturated FAs are further categorized in to monounsaturated FAs and polyunsaturated according to the number of the double bonds.

The progression of metabolic diseases is not only related to dietary TG intake but also to its composition. Dietary TG is a group of compounds with various chemical properties, and the FA and TG compositions of all lipids vary significantly (Ye et al., 2019). From the perspective of intestinal TG absorption, the rate order of dietary TG digestion is commonly reported as long-chain length TG < medium-chain length TG (MCT) < short-chain length TG (Ye et al., 2019). PL cleaves the *sn*-1 and *sn*-3 sites of TG to form free FAs and 2-MAG (Figure 2A). LCFAs and 2-MAG are re-esterified to form resynthesized TG and CM, which are then secreted into the lymphatic system. In contrast, SCFAs (2–4C) and MCFAs (6–12C) are soluble in the aqueous phase of intestinal contents, absorbed in combination with albumin, and delivered to the liver through the portal vein or directly absorbed in the stomach (Thomson et al., 1989). Due to the lack of FAs for 2-MAG re-esterification, the amount of CM secretions in lymph is significantly decreased. MCT administration does not induce an apparent maximum number of CM particles in lymph. Diets containing MCFA are less likely to predispose to obesity than those containing LCFA, and MCT may be a treatment for postprandial hypertriglyceridemia (Papamandjaris et al., 1998; Folwaczny et al., 2021). Patients with metabolic disorders have recently paid wide attention to DAG. DAG does not follow the monoacylglycerol pathway for TG resynthesis. DAGs, in particular *sn-*1,3-DAG, are digested in the small intestine by lipase to synthesize *sn-*1-MAG or *sn*-3 MAG (Lee et al., 2020). Compared to 2-MAG, the re-esterification ability of *sn-*1-MAG and *sn-*3-MAG is poor. This unique metabolic pattern hinders the release of CM, producing a lipid-lowering effect (Lee et al., 2020).

The location of FAs in TG can also affect lipids absorption efficiency and metabolic syndrome (Figure 2B). Ye *et al.* demonstrated that short-chain saturated FAs at −1,3 position could favor lipid digestion. Lipids with faster digestion may affect serum lipid profiles more easily and persistently (Ye et al., 2019). In infants, the amount of double bonds (unsaturated FAs) was positively related to fat absorption (Hageman et al., 2019). However, the lipid absorption efficiency cannot be used alone to determine the effect on metabolic syndrome. Although the MCFA and MCT are easily absorbed, they are systematically associated with higher energy expenditure and are also recommended to control obesity phenotypes (Rial et al., 2016). MCFAs can significantly reduce late apoptotic and necrotic cells, decrease the levels of inflammatory markers and exert benefits in the treatment of NAFLD (Wang et al., 2016). Polyunsaturated fats are more easily absorbed than LCFAs and are inversely associated with insulin sensitivity (Bray et al., 2002).

Dietary TG can be classified into saturated, monounsaturated, and polyunsaturated fats based on the double bonds in the hydrocarbon chain. Although there are few reports on the difference in lipid absorption between saturated FAs and unsaturated FAs, both can significantly increase postprandial plasma TG (Folwaczny et al., 2021). Unsaturated FAs enriched diets such as the Mediterranean diet have been reported to have a potential anti-inflammation and anti-obesity effect. Conversely, western diets and diets rich in saturated fats accelerate the synthesis of pro-inflammatory chemokines and cytokines, contributing to the development of metabolic diseases (Ravaut et al., 2020).

## 4 Impacts of high-fat diet-induced obesity and metabolic diseases on intestinal function

### 4.1 Changes in intestinal morphology

Intestinal epithelial cells exhibit significant cell replacement and are well-adapted to different nutritional states of the body. Dietary lipids strongly stimulate mucosal regeneration (Buts et al., 1990). Short-term (3 weeks) HFD (containing 40% lipids) significantly increased the mitosis index of jejunum in B6D2F1 mice, increasing villous size and absorption area. The expression of genes related to LCFA uptake, transport, and lipoprotein synthesis is also induced during HFD feeding (Petit et al., 2007). However, after returning to the chow diet, the changes described above also returned to normal, demonstrating that the intestine has a powerful adaptation to cope with dietary fat. With the prolongation of HFD (59% kcal from fat) intake, Swiss mice fed with a HFD for 8 weeks showed a decreased length of the small intestine and decreased circumference of the ileum. In the small intestine of mouse, morphometric parameters, including the villi, crypts, muscular layer, and total wall were decreased in the distal part but increased in the proximal part (Soares et al., 2015). When the C57/BL6 mice were fed a HFD (60% kcal from fat) for 14 weeks, the length of villi in the small intestine and the depth of crypts in the colon were significantly shorter than those in control. The 14-week HFD led to increased intestinal stem cell counts, apoptosis rate, and decreased goblet cell numbers and mucin levels (Xie et al., 2020).

The intestinal epithelial basement membrane prevents CMs from randomly entering the lamina propria. During acute HFD, CM leaves the intestinal cells and accumulates in the intercellular space, putting pressure on the tight connections between cells. The basement membrane may rupture due to stretching, resulting in increased mucosal permeability and facilitating the entry of CMs and other harmful substances into the lamina propria (Zhou et al., 2020). The exact mechanism of CM transport from basolateral membrane requires further investigation. Nevertheless, the leaky gut and impaired tight connections between cells provide a better understanding of HFD-induced intestinal barrier damage and metabolic disorders caused by intestinal dysfunction.

Intestinal neuron loss was found in the duodenum of mice fed a HFD (72% kcal from fat) for 8 weeks (Stenkamp-Strahm et al., 2013). According to statistics, the proportion of obese people in patients with gastroparesis can reach 29% (Parkman et al., 2021). The hospitalization rate of obese gastroparesis patients accounted for 13.75% of the total gastroparesis patients (Dahiya et al., 2022). Neuron loss-induced changes in gastrointestinal motility also contribute to the development and maintenance of obesity. Moreover, intestinal blood flow can be up to twice as high after dietary lipid ingestion than after fasting, a phenomenon called postprandial congestion. LCFAs are more effective than other nutrients in inducing hyperemia in the distal small intestine (Eduardo, 2005). Increased intestinal blood flow enhances free FA extraction from circulation into enterocytes, providing mitochondrial oxidation substrates and influencing intestinal metabolism. Recently, Koffert *et al.* reported that obesity could not be normalized by bariatric surgery. Although postoperative patients lost 24% of their weight, they still exhibited increased FA uptake and unchanged duodenal blood flow (Koffert et al., 2018).

Intestinal epithelial cells respond to lumen stimulation by producing chemokines and cytokines that induce the accumulation of lamina propria immune cells in areas of inflammation. Dietary nutrients, especially lipids, can have side effects that trigger immune cell activation. More free FAs derived from a HFD are cytotoxic and can disrupt the intestinal immune system [reviewed in (Basson et al., 2020)]. Tamala *et al.* demonstrated that reduced numbers of small intestinal intraepithelial lymphocytes and lamina propria lymphocytes could be found in mice fed HFD for only 1-day. HFD feeding for 3 weeks induced atrophy of gut-associated lymphoid tissue (Tanaka et al., 2020). In DSS-induced colitis mice, HFD aggravates experimental colitis by decreasing the number of goblet cells and mucin levels (Lee et al., 2017). In clinical patients, a large amount of animal lipid intake plays a role in the pathogenesis of inflammatory bowel disease (Ji et al., 2011). In addition, dietary fat-activated mast cells release mucosal mast cell protease II (RMCPII), which directly increases the permeability of epithelial cells by reducing the expression of tight junction-related proteins (Scudamore et al., 1998). RMCPII can also selectively attack type IV collagen, a vital component of the basal membrane (Scudamore et al., 1998). The activation of mucosal mast cells may be involved in intestinal permeability and insular membrane incompleteness, facilitating fat transport and uptake (Ji et al., 2012).

### 4.2 Changes in dietary nutrient absorption

Dietary TG is a potent suppressor of further energy intake, but chronic HFD reduces the satiating effect of enteral oleic acid infusion (Covasa and Ritter, 1999). In obese rats, chronic HFD feeding leads to increased gastric emptying, partly due to impaired cholecystokinin signaling (Covasa and Ritter, 2000). In human studies, obesity is also closely associated with rapid gastric emptying and fasting gastric volume (Acosta et al., 2015). Although obese patients obtain the overload energy intake from the diet, increased gastric emptying causing decreased fullness could contribute to greater energy intake (Paulino et al., 2008).

HFD consumption can directly or indirectly affect each step of TG absorption. Chronic HFD could increase the synthesis and release of pancreatic lipase, facilitating TG digestion (Sabb et al., 1986). Unlike the genes related to TG absorption in the duodenum mucosa that are decreased by a single HFD, chronic HFD can increase the expression of these genes, with the middle part of the small intestine obtaining the most pronounced effect (de Wit et al., 2008; Yoshioka et al., 2008). C57BL/6 mice fed HFD for up to 12 weeks exhibited a dramatic upregulation of LCFA uptake/trafficking (*Fatp-4*, *I-Fabp*, *L-Fabp*, *Mtp*) and lipoprotein synthesis (*ApoA-IV*) gene expression (Torelli Hijo et al., 2019).

HFD can modulate nutrient transporters that may further influence overall nutrient uptake. High-fat consumption decreases the expression of cholesterol transporter niemann-pick C1-like 1, influencing cholesterol absorption (de Vogel-van den Bosch et al., 2008). The peptide cotransporter-1 solute carrier transporter 15A1 protein, which transports dipeptide and tripeptide products of protein digestion in the small intestine, was also reduced in HFD-fed mice, suggesting that HFD-fed mice suffer from impaired peptide absorption (Do et al., 2014). Moreover, carbohydrates transporter GLUT2 content also decreased significantly (Torelli Hijo et al., 2019). Surprisingly, the downregulated expression of nutrient transporters in mice was detected in the third week of HFD, though the obesity phenotype occurred after 6 weeks (Torelli Hijo et al., 2019). Moreover, HFD also enhances sodium transporter Na (+)-H (+) exchanger 3 content, partly contributing to the hypertension progression in obesity (Torelli Hijo et al., 2019). Mariana *et al.* demonstrated that HFD feeding affects the expression of intestinal nutrient transporters in hyperthyroidism or hypothyroidism mice through an independent mechanism of PPAR-α (Losacco et al., 2018). High levels of fat intake can have a negative effect on nutritional status, introducing an obesity-related malnutrition concept.

### 4.3 Changed the cytoplasmic lipid droplets storage in enterocytes

Enterocytes can not only transport dietary TG but also act as storage organs to store the resynthesed TG in CLDs. In obesity, fat is deposited in the liver and adipose tissues. Similarly, ectopic fat deposition has also been observed in the intestine. Dietary fat intake can lead to a first increase and then decrease in the number and size of CLDs (Uchida et al., 2012). In HFD-induced obese mice and ob/ob mice, mucosa accumulated larger CLDs in the fed state (Uchida et al., 2012). *In vitro*, Caco-2/15 enterocytes, with prolonged exposure to high lipid concentrations, also exhibited CLD growth (Auclair et al., 2019). D'Aquila *et al.* found that diet-induced obese mice altered the CLD proteome, with increased or unique subgroup of lipid-related proteins involved in steroid synthesis, TG synthesis, and lipolysis (D'Aquila et al., 2019). Some candidate protein species and locations, such as perilipin family members, are also differed in the HFD model. Lee *et al.* found that perilipin family members act differently in the dietary fat challenge. Perilipin2 and perilipin3 proteins were present in the lean and dietary fat-challenged mice. Meanwhile, in acute and chronic HFD-fed mice, only perilipin2 coated CLDs in enterocytes and stabilized stored TG (Lee et al., 2009). Chronic HFD-induced obesity influences the expression of genes involved in CLD metabolism. Compared with lean mice, DIO mice had lower levels of genes related to lipolysis and fatty acid oxidation (FAO), and genes associated with TG synthesis, CM synthesis, TG storage, and lipolysis were not induced by acute dietary fat challenge (Uchida et al., 2012; D'Aquila et al., 2019). However, during the acute dietary fat challenge, DIO mice did not show increased expression of these genes. Further, the levels of genes mediating CLD hydrolysis were markedly lower 2 h after the challenge than baseline (Uchida et al., 2012).

### 4.4 Changed chylomicron secretion

An increased dietary TG load may lead to three different models of CM secretion: i) expansion of the size of CMs, ii) an increase in the number of CMs, and iii) a combination of these processes. In insulin-deficient rat model, the secretion rate of CMs is decreased, whereas the size of CMs is increased (Martins et al., 1994). To adapt to these changes in CM secretion, plasma TG clearance is enhanced in HFD mice. In HFD induced obese mice, the expression of intestinal ApoC-III gene, a potent inhibitor of blood CM clearance lipoprotein lipase, was decreased. In contrast, the expression of ApoC-II, a strong activator of lipoprotein lipase, was increased. High ApoC-II/III ratio contributes to increased clearance of CMs from the blood (Petit et al., 2007). Moreover, FAO is activated to metabolize lipids in enterocytes, preventing excess dietary TG from being transported into circulation (Kondo et al., 2006; de Wit et al., 2008). The decreased CM secretion rate and enhanced lipid oxidation and clearance function may be among the reasons for the decrease in TG levels in mice fed a TG-rich diet. However, plasma TG level changes after lipid challenge are inversely correlated with the body weight increase (Ji and Friedman, 2008). Kondo *et al.* also demonstrated that fat-mediated lipid metabolic adaptation was related to susceptibility to obesity (Kondo et al., 2006). The increasing number of CMs in response to dietary TG load may lead to increased CM remnants, resulting in an increased risk of diabetes and atherosclerosis (Tomkin and Owens, 2001). CMs and very low-density lipoproteins are the primary TG sources for peripheral cells and tissues. In the postprandial state, the number and size of CMs in the circulation increase markedly. Compared to very low-density lipoprotein, CMs are the first lipoprotein lipase substrates, allowing very low-density lipoprotein to remain in circulation for a prolonged period of time, increasing the risk of atherosclerosis (Potts et al., 1991).

## 5 Effects of the genes involved in regulating dietary triglyceride absorption on obesity and related metabolic disorders

### 5.1 Focusing on dietary triglyceride digestion

Bile acids (BAs) emulsify dietary lipids and facilitate the hydrolysis and absorption of fats in the proximal intestine. The composition of BAs can determine the absorption efficiency of dietary lipids. Mice lacking 12α-hydroxylase cytochrome P450 family 8 subfamily B member 1gene (Cyp8b1^−/−^) have altered BA composition, impaired absorption of dietary TG, increased fecal TG levels, decreased body weight and improved glucose tolerance (Bertaggia et al., 2017; Higuchi et al., 2020). The 12α hydroxylated BAs, such as cholic acid, taurocholate acid and glycocholic acid, have been reported to be associated with IR and high TG levels (Haeusler et al., 2013). Cyp8b1^−/−^ mice treated with 12α-hydroxylated BA taurocholic acid or CA restored the absorption of lipids (Bertaggia et al., 2017; Higuchi et al., 2020). However, using a bile acid sequestrant to modulate the bile acid pool is also accompanied by persistent gastrointestinal adverse reactions, such as constipation, bloating, and hypertriglyceridemia (Goldfine et al., 2010) (Table 1).

**TABLE 1:** Genetic or pharmacological manipulation of dietary lipid absorption.

Target	Gene/Protein	Genetic manipulation and phenotype of genetic model	Negative effects or limitations
*LCFA transporter*	*Cd36*	Global deletion: unchanged LCFA uptake and lipid absorption Goudriaan et al. (2002); inhibited the PCTV generation Siddiqi et al. (2010)	CD36 deficient humans: developed postprandial hypertriglyceridemia, IR and cardiovascular disease Masuda et al. (2009)
		CD36 deletion in LECs: increased button junctions Cifarelli et al. (2021)	Intestinal specific deletion: induce a leaky epithelial barrier and inflammation Cifarelli et al. (2017)
	*Fatp4*	Global deletion: enhanced glucose-dependent insulin secretion and inhibited gastric emptying Poreba et al. (2012)	Global deletion: unmodified fat absorption and cannot obtain a protective effect against obesity during HFD Shim et al. (2009)
	*Caveolin1*	Global deletion or intestinal specific deletion: steatorrhea; severely hypertriglyceridemia Razani et al. (2002)
			Global or intestinal specific deletion resistant to diet induced obesity, increased fecal fat Razani et al. (2002); Siddiqi et al. (2013)
	*I-Fabp*	Global deletion: lean phenotype Lackey et al. (2020); In male: decreased intestinal TG secretion only; greater liver mass, hepatic TG and gain more weight Agellon et al. (2007)	Global deletion: exhibit a gender dimorphic response; fragile intestinal tissue and increased intestinal barrier dysfunction; hypertriglyceridemia and IR Lackey et al. (2020)
	*L-Fabp*	Global deletion: obesity phenotype with lower liver TG content, normal blood glucose, blood lipids, lower intestinal TG secretion rate Gajda et al. (2013); Lackey et al. (2020); decreased PCTV budding activity Siddiqi et al. (2010) or protected against obesity and hepatic steatosis in HFD Newberry et al. (2006)	Global deletion: some contrast results of different changes of body weight
*TG resynthesis*	*Acsl5*	Global deletion: lower fat mass, lower blood TG and glucose levels increased insulin sensitivity Bowman et al. (2016) unchanged LCFA absorption and body weight Meller N et al. (2013)	*Acsl5* variant in the neonatal period: failure to thrive Al-Thihli et al. (2021)
			Deficiency of *Acsl5* in adult: impaired intestinal epithelium Meller N et al. (2013)
	*Mgat2*	Intestinal specific deficiency: delayed lipid absorption; increased energy expenditure; improved insulin sensitivity and protect HFD induced obesity Gao et al. (2013); Nelson et al. (2014)	MGAT2 inhibitors: minor gastrointestinal adverse reactions, and have a good effect on the treatment of obesity
	*Dgat1*	Global or intestine specific deletion: delayed lipids absorption; increased lipid accumulation in intestine; improved leptin and insulin sensitivity protect HFD induced obesity Chen et al. (2002)	DGAT1 inhibitors: diarrhea, intestinal cytotoxic FA
			DGAT1 variant: intestinal failure, life-threatening diarrhea and nutrient malabsorption van Rijn et al. (2018)
	*Dgat2*	Intestine-specific overexpression: higher TG secretion rate, hypertriglyceridemia and increased susceptibility to hepatic steatosis Buhman et al. (2002); van Rijn et al. (2019)	Global deletion: die after birth due to too little fat and severely impaired skin permeability barrier function Stone et al. (2004)
*lipids pool: CLDs formation*	Perilipin2	Global deletion: decreased intestinal CLD content, increased fecal TG content; reductions in food intake protect HFD induced obesity, NAFLD Frank et al. (2015); Xiong et al. (2017)	Perilipin2 loss exhibits protective effects on diet-induced obesity rely on sufficient time (>4W) of exposure to HFD McManaman et al. (2013)
	*Lpcat3*	Intestine-specific deletion: increased enterocyte lipid accumulation and decreased lipid absorption Li et al. (2015); Kabir et al. (2016)	Global deletion: neonatal lethality
*lipids pool: CLDs breakdown*	*Atgl*	Intestine-specific deletion: increased CLDs in enterocyte but unchanged TG absorption, plasma lipid parameters and body weight Obrowsky et al. (2013)	Intestine-specific deletion: delayed cholesterol absorption
	*Cgi-58*	Intestine-specific deletion: increased CLD accumulation in small intestine; inefficient CMs secretion; lower plasma TG concentration; and attenuated hepatic steatosis Xie et al. (2014); Korbelius et al. (2019)	Mutations in human CGI-58: Chanarin-Dorfman Syndrome
			Global deficiency: a permeability barrier defect of the skin and mice die shortly after birth
	*Mgl*	Intestine-specific overexpression: increase in body weight gain, increased hepatic and plasma TG levels Chon et al. (2012)	MGL inhibitor: inhibited whole gut transit
		Global deletion: delayed fat absorption, decreased body weight gain and prevented hepatic steatosis Tardelli et al. (2019); Yoshida et al. (2019)	Global deletion: reduced plasma free FA levels, tend to develop hypoglycemia, and stay in torpor-like state during prolonged fasting Schoiswohl et al. (2010)
*CMs assembly*	*Mtp*	Intestine-specific deletion: reduced CMs secretion, increased accumulated TG in enterocytes, improved of hepatic steatosis and insulin sensibility without weight loss Xie et al. (2006); Xie et al. (2021)	MTP inhibitor: GI tolerability (diarrhea); targets both intestinal and hepatic MTP might lead to steatohepatitis and fibrosis
			*MTP* gene mutation: malnutrition and abetalipoproteinemia
			Intestine-specific deletion: impaired intestinal barrier function
	*ApoB-48*	Functional intestine-specific down expression: impaired CMs production and lipids absorption and prevented obesity and fatty liver Gaskell et al. (2020)Anti-ApoB 48 antibodies: inhibited the PCTV generation Siddiqi et al. (2010)	Defective translation of full length ApoB: familial hypobetalipoproteinemia and more sensitive to obesity and NAFLD Di Filippo et al. (2014)
		Global deletion: reduced plasma TG and cholesterol levels; decreased lipid absorption efficiency without malabsorption of lipids Kohan et al. (2012)	
	*ApoA-IV*	Global deletion: reduced plasma TG and cholesterol levels; decreased lipid absorption efficiency without malabsorption of lipids Kohan et al. (2012)
	*ApoC-III*	Global overexpression: inhibits CMs secretion; and mice exhibits fewer and smaller CLDs; decreased plasma TG Li D et al. (2019)	Elevated ApoC-III level is an independent risk factor for atherogenic
*CMs trafficking*	*Sar1b*	Global overexpression: increase intestinal fat absorption, and mice exhibit obesity and liver steatosis phenotype Levy et al. (2014) deletion of SAR1B *in vitro*: cannot completely eliminate CMs secretion Sane et al. (2017)	*SAR1B* gene mutation was related to disorder chylomicron retention disease
*CMs passaging through lamina propria*	*Dennd5b*	Global deletion: impaired CMs transporting out of enterocytes, resistant to western diet induced obesity and NAFLD, improved plasma lipids and reduced aortic atherosclerosis; Variant allele homozygous: Females had significantly lower BMI and abdominal circumference Gordon et al. (2019)	In human, homozygous deletion of functional variation results in a highly deleterious phenotype
	*Plagl2*	Global deletion: grow arrest or even died shortly after birth due to starvation Van Dyck et al. (2007)
			Global deletion CMs fail to exit the gut interstitium and mice exhibit severe emaciation Van Dyck et al. (2007)
*Entering into Lacteals*	*Vegfc*	Genetical deletion	During perinatal period, VEGF-C deletion induce embryos die due to arrested lymphangiogenesis Nurmi et al. (2015)
		Lacteal regression without affecting other lymphatic beds, decrease lipid absorption and protect HFD induced obesity Nurmi et al. (2015)	
	*Vegfr3*	*Chy* mouse model (inactivated VEGFR-3): zippering of lacteal junctions, increased TG accumulation in enterocytes, increased fecal TG content, reduced postprandial plasma TG levels and metabolic protective Shew et al. (2018)	*Chy* mice develop lipid-rich chylous ascites at birth
	VEGF-A	Increased VEGF-A bioavailability and signaling through VEGFR2 (Global deletion of VEGFA receptors): Zippering of lacteal junctions, improved glucose tolerance and dyslipidemia, protected from diet induced obesity and NAFLD Zhang et al. (2018)	Increased VEGF-A bioavailability: increase permeability in blood vascular Zhang et al. (2018); correlated with an unfavorable prognosis and disease severity

Abbreviations: Acsl5, long chain acyl-CoA synthetases5; ApoA-IV, Apolipoprotein A-IV; ApoB-48, Apolipoprotein B-48; ApoC-III, Apolipoprotein C-III; atgl, adipose triglyceride lipase; Cgi-58, comparative gene identification-58; CLDs, cytoplasmic lipid droplets; CMs, chylomicrons; Dennd5b, DENN, domain containing protein 5b; Dgat, diacylglycerol acyltransferase; Fatp4, fatty acid transport protein 4; HFD, high-fat diet; I-Fabp, intestinal fatty acid binding protein; IR, insulin resistance; LCFA, long chain fatty acid; L-Fabp, liver fatty acid binding protein; Lpcat, lysophosphatidylcholine acyltransferases; Mgat, monoacylglycerol acyltransferase; Mgl, monoacylglycerol lipase; Mtp, microsomal triglyceride transfer protein; NAFLD, non-alcoholic fatty liver disease; PCTV, pre-chylomicron transport vesicle; Plagl2, pleomorphic adenoma gene-like 2; Sar1b, Ras-related GTPase 1B; TG, triacylglycerol; VEGF, vascular endothelial growth factor; VEGFR-3, Vascular endothelial growth factor receptor 3.

PL is responsible for the hydrolysis of 50%–70% of dietary lipids (Armand, 2007; Birari and Bhutani, 2007). PL inhibitors influence the root cause of obesity (Kumar and Chauhan, 2021). Orlistat, a well-known PL inhibitor, is the only authorized anti-obesity drug (McClendon et al., 2009). However, the use of orlistat is associated with gastrointestinal adverse effects, such as oily stools and diarrhea (Filippatos et al., 2008). Apart from Orlistat, plenty of phytoconstituents, including flavonoids, saponins, alkaloids, and terpenoids, also have profound inhibitory effects on PL (Rajan et al., 2020; Kumar and Chauhan, 2021). Though blocking fat digestion is an exciting opportunity for treating obesity, the unabsorbed lipid transport into the lower intestine can activate GPR119 to slow gastric emptying (Higuchi et al., 2020).

### 5.2 Focus on long-chain fatty acid uptake and traffic in enterocytes

#### 5.2.1 Long-chain fatty acid uptake

Intestinal absorption of LCFA requires CD36. *Cd*36 gene deficiency (Cd36^−/−^) mice showed a 50% reduction in FA uptake in proximal enterocytes (Nassir et al., 2007). However, based on fecal lipid content and blood appearance, Cd36^−/−^ mice showed no evidence of reduced lipid absorption. They only had a reduced rate of TG entry into lymph and serum (Goudriaan et al., 2002). The unaltered lipid uptake in Cd36^−/−^ mice may be due in part to ultimate compensation by the transfer of the lipid absorption sites to the distal parts of small intestine (Nassir et al., 2007). However, CD36 also influences the catabolism of TG-rich CMs in plasma. In Cd36^−/−^mice, lipoprotein lipase activity was normal, but the clearance of intestine-derived lipoproteins was slow (Drover et al., 2005). In addition, people with *CD36* deficiency develop postprandial hypertriglyceridemia, IR, and cardiovascular disease through enhanced lipoprotein remnant levels in plasma (Masuda et al., 2009). Hajri *et al.* demonstrated that global Cd36^−/−^ mice were protected against obesity, mainly caused by impaired FA sensing by adipocytes and elevated leptin levels (Hajri et al., 2007). However, *Cd36* deletion in endothelial cells result in a leaky epithelial barrier and inflammation (Cifarelli et al., 2017).

In the FATP family, FATP4 is mainly expressed in jejunum (Buttet et al., 2014). *In vivo*, isolated enterocytes with a 48% reduction in FATP4 protein showed a 40% reduction in FA uptake (Gimeno et al., 2003). Nevertheless, *Fatp*4 deletion (Fatp4^−/−^) mice exhibited no modified fat absorption and had no protection against obesity during HFD feeding (Shim et al., 2009). However, FATP4 is not ineffective in regulating metabolic disorders. For example, *Fatp4* deletion plays a crucial role in oleic acid-induced GLP-1 secretion, enhancing glucose-dependent insulin secretion and inhibiting gastric emptying (Poreba et al., 2012).

Caveolar vesicle structures are formed by the caveolin proteins, which are present in a single caveola and can directly bind to FAs (Trigatti et al., 1999; Parton and Simons, 2007). Global *Caveolin-1* deletion (Cav1^−/−^) mice showed protection against diet-induced obesity, partly due to the higher presence of steatorrhea (Razani et al., 2002; Siddiqi et al., 2013). However, serum TG, cholesterol, and free FA levels were severely elevated in Cav1^−/−^mice, especially in the postprandial state (Razani et al., 2002). Intestinal specific-*Caveolin-1* deletion (Cav1^iEC−KO^) mice decreased circulating free FAs and low-density lipoproteins cholesterol levels, preventing HFD-induced obesity and rapid increase in low-density lipoproteins cholesterol (Otis et al., 2017). Moreover, global Cav1^−/−^ mice exhibited elevated very low-density lipoproteins and low-density lipoproteins cholesterol levels, mainly resulting from reduced lipid storage by adipocytes and hepatic lipoprotein cholesterol uptake (Frank et al., 2008). The increased circulating free FA levels in Cav1^iEC−KO^ mice may be due to decreased hepatic FAs instead of altered intestinal processing (Otis et al., 2017).

#### 5.2.2 Long-chain fatty acid traffic

FAs are transported to specific metabolic sites within enterocytes by FABPs. *In vitro*, both L-FABP and I-FABP are involved in FA uptake from the intestinal lumen and circulating blood into enterocytes (Alpers et al., 2000). The function of the two FABPs is independent because eliminating one gene does not cause compensatory upregulation of the other (Storch and Thumser, 2010). *I-Fabp* deletion (I-Fabp^−/−^) mice exhibited a leaner phenotype when fed a HFD than wild-type mice, whereas *L-Fabp* deletion (L-Fabp^−/−^) mice displayed higher body weight and body fat mass (Gajda et al., 2013; Lackey et al., 2020). There are also some contrasting results. Newberry *et al.* found that L-Fabp^−/−^ mice were protective against western diet-induced obesity and hepatic steatosis (Newberry et al., 2006). During HFD feeding, male I-Fabp^−/−^ mice had greater liver mass and gained more weight than wild-type mice. However, female I-Fabp^−/−^ mice exhibited smaller livers and lower weight gain (Agellon et al., 2007). These different phenotypes may result from various factors such as lipid species in the HFD, sex, residual background genes, and even living environment (Newberry et al., 2015). Although L-Fabp^−/−^ mice exhibited different changes in body weight, lower liver TG content and decreased FAO were consistent (Newberry et al., 2006). L-Fabp^−/−^ obese mice had normal blood glucose and lipid levels, lower intestinal TG secretion rate, and greater exercise tolerance (Gajda et al., 2013).

The leaner phenotype of I-Fabp^−/−^ mice is not dependent on fat malabsorption. Instead, the shortened movement time of the small intestine and blunt villi lead to changes in nutrient absorption, thus affecting systemic energy metabolism (Lackey et al., 2020). Notably, the intestinal tissue in I-Fabp^−/−^ mice was more fragile, with thinner muscular layer and higher permeability in the proximal small intestine. I-Fabp^−/−^ mice exhibited intestinal dysbiosis and even pathophysiological changes under HFD feeding (Lau et al., 2016; Lackey et al., 2020). In humans, *L-FABP* is more abundant than *I-FABP*. The *I-FABP* mutation can increase the affinity to FA, which is associated with hypertriglyceridemia and IR in Pima Indians (Baier et al., 1995).

### 5.3 Focus on the intestinal triglyceride resynthesis

#### 5.3.1 Long-chain fatty acid-coenzyme A synthesis

ACSL catalyzes the first step of TG resynthesis. ACSL5 and ACSL3 are the main synthetases in the intestine and ACSL5 contributes 60%–80% of the total activity of intestinal ACSL activity (Meller N et al., 2013). ACSL5 transports FAs for lipid biosynthesis. During the neonatal period, *Acsl5* variant is associated with recurrent vomiting, diarrhea, and failure to thrive (Al-Thihli et al., 2021). The administration of MCT that is absorbed directly through the portal vein or stomach can attenuate the aforementioned adverse effects, reflecting the importance of ACSL5 in promoting long-chain length TG absorption (Al-Thihli et al., 2021). Global *Acsl5* deletion (Acsl5^−/−^) mice exhibited decreased TG absorption, lower fat mass, lower blood TG and glucose levels, and increased insulin sensitivity without acute postnatal complications (Bowman et al., 2016). Meanwhile, dietary LCFA absorption and body weight gain remained unaffected in Acsl5^−/−^ mice, possibly due to the residual ACSL activity that can maintain normal LCFA absorption (Meller N et al., 2013). However, *Acsl5* deficiency may impair intestinal epithelium. Reduced *Acsl5* levels can lead to high concentrations of ceramide-mediated apoptosis and oxidative stress of unesterified FAs in intestinal epithelial cells (Al-Thihli et al., 2021). *Acsl3* gene expression is induced by the liver X receptor, and liver X receptor agonists promote *Acsl3* expression and delay lipid secretion in zebrafish (Cruz-Garcia and Schlegel, 2014).

#### 5.3.2 DAG synthesis

In the MGAT family, MGAT2 is highly expressed in the intestine of human and mouse, and MGAT3 is only highly expressed in the distal small intestine of human. Mice without global *Mgat2* genes (Mgat2^−/−^) exhibited resistance to HFD-induced obesity, although they consumed and absorbed normal amounts of dietary fat (Yen et al., 2009). In global Mgat2^−/−^ mice, intestine-specific overexpression of *Mgat2* restored fat absorption rate and metabolic efficiency and promoted weight gain during HFD (Gao et al., 2013). In addition, mice with intestinal-specific *Mgat2* deficiency exhibited similar delayed fat absorption and increased energy expenditure. They were equally resistant to HFD-induced NAFLD, hypercholesterolemia, and glucose intolerance (Nelson et al., 2014). In addition, global Mgat2^−/−^ mice showed increased GLP-1 and PYY secretion, which contributed to appetite reduction and glycemic control (Yen et al., 2009). However, global Mgat2^−/−^ mice fed a fat-free diet also showed increased energy expenditure, which suggested that MGAT2 modulates energy expenditure and may be independent of dietary fat absorption (Nelson et al., 2011).

Recently, some small-molecule MGAT2 inhibitors with significant structural diversity may be used to treat obesity and metabolic diseases further (Devasthale and Cheng, 2018). However, MGAT2 inhibitors result in minor gastrointestinal adverse reactions that may be related to the unique presence of MGAT3 in the human intestine. Although the role of MGAT3 in lipid absorption has been recognized for a long time, this gene is lacking in the intestinal tract of mice and other lower animals. The construction of an appropriate animal model can help further understand the physiological functions of MGAT3.

#### 5.3.3 Triglyceride resynthesis

DGAT enzyme is encoded by two non-homologous genes, DGAT1 and DGAT2 (Yen et al., 2008). Deletion of the *Dgat1* gene (Dgat1^−/−^) increased energy expenditure in mice, improved leptin and insulin sensitivity, and improved resistance to diet-induced obesity and NAFLD (Chen et al., 2002). In addition, in lipid stress testing and HFD feeding studies, gut-specific deletion of *Dgat1* gene or DGAT1 inhibitors led to increased accumulation of total neutral lipid droplets in proximal intestinal epithelial cells and the delayed release of CMs into the blood (Ables et al., 2012; Maciejewski et al., 2013; Hung and Buhman, 2019). Notably, after restoring the function of intestinal DGAT1 in Dgat1^−/−^mice, the resistance to HFD-induced hepatic steatosis and obesity disappeared (Lee et al., 2010).

Studies have also suggested that the effect of DGAT2 on intestinal fat absorption is greater than that of DGAT1 (Buhman et al., 2002). DGAT2 is necessary for the growth and development of mice, as *Dgat2* gene deletion mice die after birth due to insufficient fat and severely impaired skin permeability barrier function (Stone et al., 2004). In DGAT1-deficient human intestinal stem cell-like organoids, DGAT2 overexpression partially compensated for CLD formation and protected against oleic acid-induced toxicity (van Rijn et al., 2019). Furthermore, *Dgat2* overexpressed mice exhibited higher CM secretion rates, contributing to hypertriglyceridemia and hepatic steatosis (Uchida et al., 2013). Since only *DGAT1* is highly expressed in the human small intestine, and *DGAT2* is expressed only at very low levels, *DGAT2* is defined as non-functional. *DGAT1* mutation is associated with congenital diarrheal disorders, intestinal failure, and aberrant lipid metabolism (van Rijn et al., 2018). DGAT1 inhibitors can lead to severe gastrointestinal reactions, such as diarrhea and nausea. The intestinal cytotoxic FA produced by DGAT1 inhibitors in humans is similar to the inhibition of DGAT1 and DGAT2 simultaneously in mice (Denison et al., 2014; van Rijn et al., 2018).

### 5.4 Focus on lipid pools

#### 5.4.1 Formation of cytoplasmic lipid droplets

**FIGURE 3:**
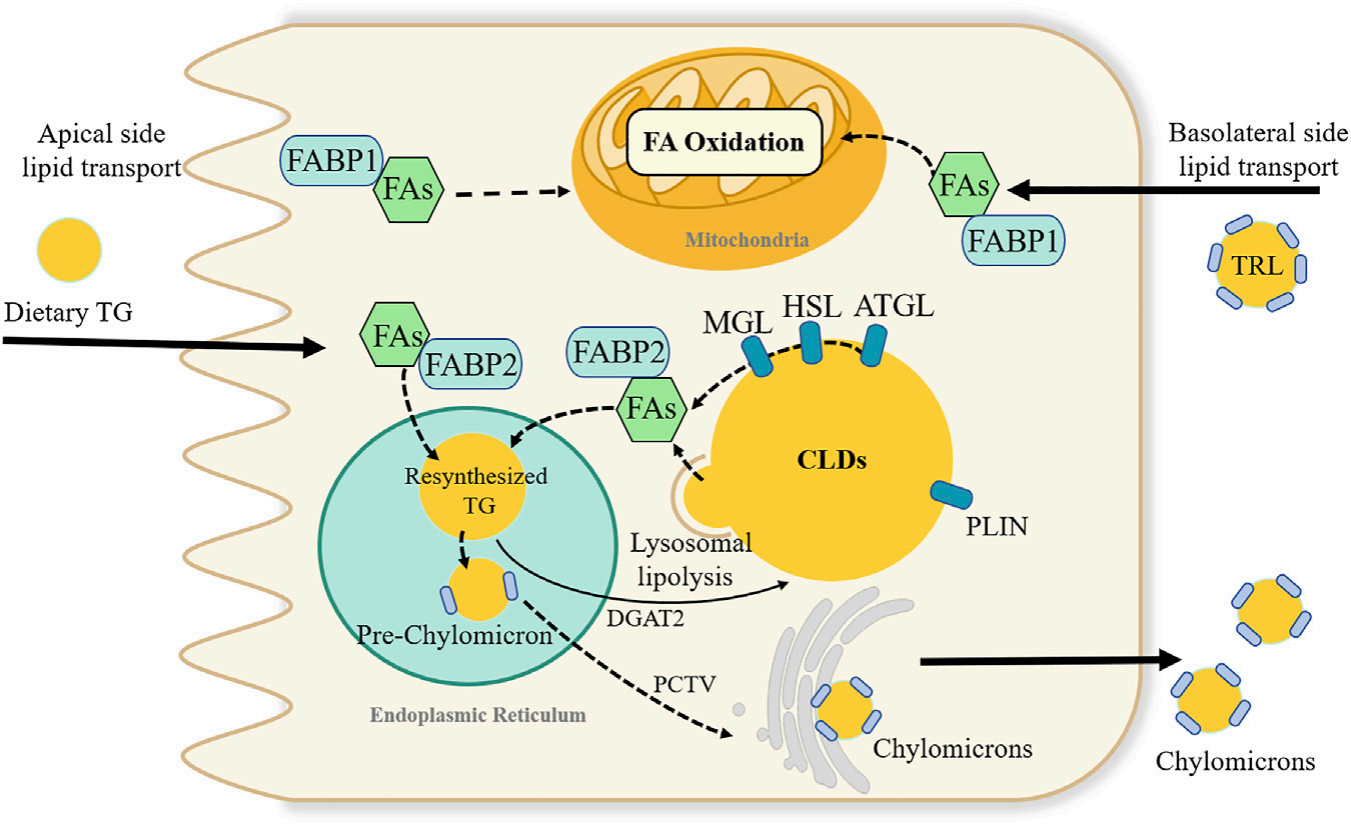
Dynamic lipids pool and proposed fatty acids metabolic fate in intestinal enterocyte. Fatty acids (FAs) in enterocytes can source from the apical side of enterocytes or the basolateral side. FAs absorbed from the intestinal lumen preferentially direct the triglyceride (TG) synthesis and undergoes secretion into lymph in chylomicrons. While, FAs from the circulatory system, i.e., basolateral side source of FAs are pre-utilized for phospholipid synthesis and FA oxidation. FAs binding to fatty acid-binding protein 1 is suggested to transported FAs towards the FA oxidation pathway. Many proteins like perilipin family involve in the stabilizing and swelling of cytoplasmic lipid droplets (CLDs) TG stored in CLDs can be break down by a series of lipases, or through lysosomal lipolysis pathway. Adipocyte triglyceride lipase (ATGL), hormone sensitive lipase (HSL) and monoacylglycerol lipase (MGL); diacylglycerol acyltransferase (DGAT2); PCTV; triglyceride-rich lipoproteins (TRL); perilipin (PLIN).

The number of CLDs in enterocytes synthesizes a dynamic lipid pool (Figure 3). Diverse proteomics have novel roles in regulating the balance of TG storage in CLDs or secretion in CMs (D'Aquila et al., 2019). More than 180 proteins are associated with the CLD fraction, among which the perilipin family has been widely reported (D'Aquila et al., 2015). In the perilipin family, perilipin2 is highly expressed in the small intestine. A global absence of the perilipin2 protein (Plin2-null) prevented HFD-induced obesity, IR, and liver steatosis, partly by limiting energy intake (McManaman et al., 2013). Plin2-null mice had increased fecal TG levels and decreased enterocyte CLD content in a 4-day HFD feeding experiment (Frank et al., 2015). Moreover, the metabolic benefits of perilipin2 deletion may also be due to increased energy expenditure or altered intestinal structure and function (Frank et al., 2015; Xiong et al., 2017).

CLD membrane is another candidate that can influence the size of CLDs. Phosphatidylcholine (PC) is a prominent phospholipid found in CLD membranes. Lysophosphatidylcholine acyltransferases3 (LPCAT3) are involved in the re-acylation of PC in the intestine (Zhang et al., 2021). Intestine-specific *Lpcat3* deficient mice exhibited enterocyte lipid accumulation and significantly reduced lipid absorption and circulating lipids and lipoproteins, which may be due to decreased protein levels of CD36 and FATP4 protein (Li et al., 2015; Kabir et al., 2016). Although whole *Lpcat3* deficiency can cause neonatal lethality, which can be saved by PC/olive oil supplementation, some survivors showed larger small intestines with shorter and wider villi. Moreover, acute liver-specific *Lpcat3* deletion exacerbates lipid-induced ER stress and hepatic steatosis (Rong et al., 2015).

Lipid fusion between CLDs facilitates their growth. Cell death-inducing DNA fragmentation factor alpha-like effector (CIDE) family proteins, which are enriched at CLD-CLD contact site, mediate the transfer of lipids from small CLDs to large CLDs (Chen et al., 2020). In the CIDE family, CIDEB is low expressed in the intestine but high expressed in the liver. Mice with global deletion of *Cideb* (Cideb^−/−^) had lower levels of plasma TG, improved IR and protection against HFD-induced obesity and fatty liver (Li et al., 2007). In the intestine, Cideb^−/−^ mice exhibited impaired CM secretion and increased CLD accumulation in enterocytes. Moreover, overexpression of *Cideb* can increase TG secretion, indicating that CIDEB may also be involved in intestinal CM lipidation (Zhang et al., 2014).

#### 5.4.2 Cytoplasmic lipid droplets breakdown

Cytoplasmic hydrolysis involves three sequential enzymes: ATGL and its coactivator comparative gene identification-58 (Cgi-58), hormone-sensitive lipase, and monoacylglycerol lipase (MGL). First, ATGL hydrolyzes TG to FA and DAG. Intestinal-specific deletion of *Atgl* (Atgl^iKO^) increases CLDs in enterocytes but does not influence TG absorption, suggesting that CM secretion might be ATGL-independent (Obrowsky et al., 2013). During HFD feeding, Atgl^iKO^ mice had similar plasma lipid parameters and body weights to those of wild-type mice. Small intestinal-specific overexpression of *Atgl* mice showed a slight elevated enzymatic activity and unchanged CM secretion (Korbelius et al., 2022). In comparison, mice deprived of *Cgi-58* in the intestine exhibited inefficient CM secretion, reduced plasma TG concentration, and increased CLD accumulation in the first proximal segment of 5 equal segments of the small intestinal, even during fasting conditions (Xie et al., 2014). Intestine-specific *Cgi-58*/*Atgl* double knockout mice exhibited increased lipid accumulation, but luminal lipid absorption and TG secretion rates were unaffected (Korbelius et al., 2019). The synthesis of CMs seems to bypass ATGL/Cgi-58 mediated catabolism. CLDs accumulated in enterocytes 2 h post-gavage of lipids, which is more likely to be caused by circulating lipid uptake, leading to decreased plasma TG (Korbelius et al., 2019). Consistently, both *Cgi-58* single-knockout mice and *Cgi-58*/*Atgl* double-knockout mice exhibited attenuated hepatic steatosis (Xie et al., 2014; Korbelius et al., 2019). ATGL plays a vital role in the lipolysis to provide energy, so Atgl-deficient mice are prone to hypoglycemia. During prolonged fasting, Atgl-deficient mice stay in a torpor-like state (Schoiswohl et al., 2010).

Hormone-sensitive lipase catalyzes the conversion of DAG to FA and MAG. Interestingly, mice with intestine-specific hormone-sensitive lipase deletion did not display impaired TG metabolism but had increased plasma cholesterol concentrations and cholesteryl ester accumulation in the small intestine, suggesting that hormone-sensitive lipase may play a more important role in maintaining cholesterol homeostasis (Obrowsky et al., 2012).

MGL is the enzyme involved in the final step of TG lipolysis, which can hydrolyze MAG. Chon *et al.* demonstrated that the primary function of MGL was to convert dietary MGs to TG instead of CLD degradation in the intestine (Chon et al., 2012). Intestinal-specific *Mgl* overexpression (iMgl) mice exhibited unaltered dietary fat absorption. Body weight, hepatic and serum TG levels of iMgl mice were higher than those of wild-type mice after only 3 weeks of HFD feeding. This may be due to the decreased levels of 2-arachidonyl glycerol caused by *Mgl* overexpression, which led to the hyperphagic behavior and decreased energy expenditure (Chon et al., 2012). Global *Mgl* deletion mice showed delayed intestinal fat absorption, prolonged TG retention in the ileum, decreased body weight gain, better IR and prevented hepatic steatosis (Tardelli et al., 2019; Yoshida et al., 2019). Global deletion of *Mgl* affected entire energy homeostasis, which was partly due to higher levels of 2-arachidonyl glycerol-induced central orexigenic stimuli (Taschler et al., 2011).

Lysosomal acid lipase is a key enzyme in the lysosomal TG lipolysis pathway. In mice, lysosomal acid lipase deficiency leads to a large accumulation of TG in the small intestine, severe fat malabsorption, and steatorrhea (Du et al., 2001). Lipophagy is a form of macroautophagy initiated by phagocytosis of CLDs by autophagosomes, but the large size of CLDs impairs its recruitment, leading to partial lipophagy of CLDs. The underlying recruitment mechanisms for CLD fragmentation and specific anchoring targets on CLD remain undefined. However, inhibition of the fusion of autophagosomes with lysosomes or lysosomal acid lipase can accumulate CLDs in Caco-2 cells (Khaldoun et al., 2014). In an *in vivo* study, DGAT1^−/−^ mice were resistant to diet-induced obesity, in part due to disruption of lysosomal function, which in turn induced abnormal CLD accumulation in enterocytes. Although there were no fat absorption disorders in DGAT1^−/−^ mice, TG secretion was slow (Hung and Buhman, 2019).

#### 5.4.3 Cytoplasmic lipid droplets pool

Synthesis of CLDs can temporarily store dietary lipids in epithelial cells, attenuate postprandial lipid fluctuations, and protect against the cellular lipotoxicity caused by the large influx of dietary FAs (Schaffer, 2003). Lipids of basolateral origin can also play a role in adjusting lipidemia. Enhanced basolateral lipid substrate transport in the gut can reduce circulating lipids and promote intestinal FAO, thus playing a protective role against diet-induced obesity (Li D et al., 2019). However, basolateral lipids contribute to FAO in intestinal cells and effectively maintain diet-derived CMs secretion.

### 5.5 Focus on chylomicron assembly and trafficking

#### 5.5.1 Chylomicron assembly

In ER lumen, MTP mediates lipidation of ApoB-48 to form pre-CM particles. Mice with conditional *Mtp* knockout in villus enterocytes (Mtp^iKO^) showed impaired CM assembly, increased accumulation of TG in enterocytes, and no CM particles in the secretory pathway. However, although Mtp^iKO^ mice exhibited decreased plasma and hepatic TG content, hepatic lipogenesis and low-density lipoproteins secretion increased, suggesting an unexpected interaction between intestinal and hepatic lipid metabolism (Xie et al., 2006). Xie *et al.* also found that Mtp^iKO^ mice fed a high-fat-fructose-cholesterol diet for 10 weeks exhibited a sustained reduction in hepatic TG (Xie et al., 2021). MTP inhibitor lomitapide, has also shown beneficial effects in improving obesity, blood glucose, lipids, and blood vessels (Hooper et al., 2015; Munkhsaikhan et al., 2022). Lomitapide, however, targets both intestinal and hepatic MTP. Therefore, it can lead to lipid accumulation in the liver and contribute to the progression of NAFLD and even fibrosis (Liu et al., 2017). Intestine-specific MTP inhibitor JTT-130 avoids lipid deposition in the liver and plays in a diet-dependent manner to reduce food intake, reduce body weight, and increase insulin sensitivity (Hata et al., 2011). In hepatic steatosis and fibrosis, blockage of CM assembly by Mtp^iKO^ can increase intestinal permeability, increase hepatic lipogenesis, and delay the remission of liver inflammation and fibrogenic signals (Xie et al., 2021). Patients with *MTP* gene mutations are characterized by an inability to produce CMs in the intestine, resulting in malnutrition and abetalipoproteinemia (Berriot-Varoqueaux N, 2000).

ApoB-48 is encoded by the *ApoB* gene and edited by Apobec-1 after transcription to generate a 48% of the length of ApoB-100 protein. Given that ApoB exists as ApoB-100 in the liver, defective translation of full-length ApoB leads to defective assembly of ApoB-containing lipoproteins from both enterocytes and hepatocytes, which is associated with familial hypobetalipoproteinemia. Patients with familial hypobetalipoproteinemia are at higher risk for obesity and NAFLD (Di Filippo et al., 2014). Other factors, such as hepatic high mobility group box-1 in intestinal epithelial cells, can also influence ApoB-48 expression, leading to lipid accumulation in enterocytes and decreased CM formation, thereby protecting mice from obesity and fatty liver (Gaskell et al., 2020).

Exchangeable apolipoproteins can also influence metabolism. ApoA-IV is the most responsive lipoprotein to dietary lipids (Qu et al., 2019). ApoA-IV can directly interact with ApoB, and when ApoA-IV is intracellularly retained in the ER, pre-CM trafficking from the ER to the Golgi is significantly reduced, resulting in delayed ApoB secretion and decreased lipid absorption efficiency under HFD (Weinberg et al., 2012). Moreover, ApoA-IV can facilitate pre-CM expansion by stabilizing pre-CM and ApoB-48, further influencing the size of CMs (Weinberg et al., 2012). Overexpression of *ApoA-IV* can enhance lipid transport in intestinal enterocytes by increasing the size of the secreted lipoproteins (Lu et al., 2006). *ApoA-IV* knockout mice exhibited reduction in plasma TG and cholesterol levels, without lipid malabsorption (Kohan et al., 2012). *ApoA-I* deficient mice showed enhanced dietary TG absorption but accelerated clearance of postprandial TG, which could protect mice from HFD-induced hepatic lipid deposition (Karavia et al., 2012). Though *apoC-III* overexpression inhibited CM secretion, transgenic mice exhibited fewer and smaller CLDs in enterocytes and secreted smaller and less density CMs (Li D et al., 2019). Moreover, excessive *apoC-III* can inhibit intestinal uptake of TG-rich lipoproteins from the basolateral surface in a dose-dependent manner, increasing the risk of atherogenesis.

#### 5.5.2 Chylomicron trafficking

The PCTV budding complex contains ApoB-48, L-FABP, CD36, COPII and vesicle-associated membrane protein7 (VAMP7) proteins. The binding of L-FABP to the ER initiates PCTV budding. *L-Fabp* deletion mice have only 21% PCTV budding activity (Siddiqi et al., 2010). SAR1B is a GTPase required for ER movement to the Golgi apparatus of pre-CM transport vesicles. In humans, SAR1B gene mutations are related to chylomicron retention disease (Levy et al., 2014). Mice with a human SAR1B genetic defect (Sar1b^−/−^) reproduce some chylomicron retention disease features. Sar1b^−/−^ mice exhibited decreased *ApoB* and *Mtp* expression, contributing to the malabsorption of fat and decreased secretion of CM (Auclair et al., 2021). Correspondingly, Sar1b overexpression (Sar1b^+/+^) can enhance intestinal fat absorption under HFD condition. Moreover, Sar1b ^+/+^ mice showed phenotypes of dyslipidemia, obesity, and liver steatosis and were susceptible to IR (Levy et al., 2014). In Caco-2/15 cells, the deletion of *SAR1B* decreased CM output but did not completely eliminate CM secretion because *SAR1A* expression can be compensatory elevated (Sane et al., 2017).

Transmembrane proteins are also involved in PCTV formation. Anti-ApoB-48, anti-VAMP7 antibodies, and gene deletion of L-FABP and CD36 can all inhibit PCTV formation (Siddiqi et al., 2010). Anti-VAMP7 antibodies eliminated in 85% of PCTV transfer from ER to Golgi apparatus (Siddiqi et al., 2006). HFD can inhibit the expression of VAMP7, and ginger-derived nanoparticles can induce the expression of VAMP7. Sorting miR-375 into exosomes *via* VAMP7 can improve insulin sensitivity without affecting dietary fat absorption (Kumar et al., 2021). Moreover, soluble N-ethylmaleimide-sensitive factor attachment protein receptor complex participates in the process of PCTV-Golgi membrane fusion. Syntaxin-binding protein 5 is a negative regulator of soluble N-ethylmaleimide-sensitive factor attachment protein receptor, and its downregulation may be involved in the glucose-mediated mobilization of TG (Xiao et al., 2019).

### 5.6 Focus on chylomicron passaging through the lamina propria

Mature CMs are transported to the basolateral membrane *via* budding CM secretory vesicles, secreted into the intercellular space by exocytosis, and further into the lamina propria. In DENN domain-containing protein 5b knockout (Dennd5b^−/−^) mice, CM transport out of enterocytes was impaired, and massive CM secretory vesicles were accumulated in the enterocytes, as CM secretory vesicles from the Golgi failed to fuse with the basolateral membrane. Dennd5b^−/−^ mice were resistant to western diet-induced obesity, exhibited improved plasma and hepatic lipid concentrations and reduced aortic atherosclerosis (Gordon et al., 2019). However, Mobilia *et al.* also demonstrated that the significant improvement of metabolic disorder in global Dennd5b^−/−^ mice were also mediated by downregulated hepatic lipid metabolism genes (Mobilia et al., 2022). Therefore, future studies in cell-specific knockout mice are needed to distinguish the beneficial metabolic effects of Denn5b in different tissues. Furthermore, pleomorphic adenoma gene-like 2 (*Plagl2*) regulates lipid entry into the lacteals. *Plagl2* deletion mice develop severe emaciation due to the blockage of CMs from the lamina propria into the lacteals, and exhibit growth arrest and even die of starvation shortly after birth. *Plagl2* null enterocytes can assemble and secrete CMs, but cannot exit the gut interstitium. PLAGL2 is involved in CM modification and is necessary for subsequent CM uptake (Van Dyck et al., 2007).

### 5.7 Focus on lymphatic lipid transport

#### 5.7.1 Lacteal survival

**FIGURE 4:**
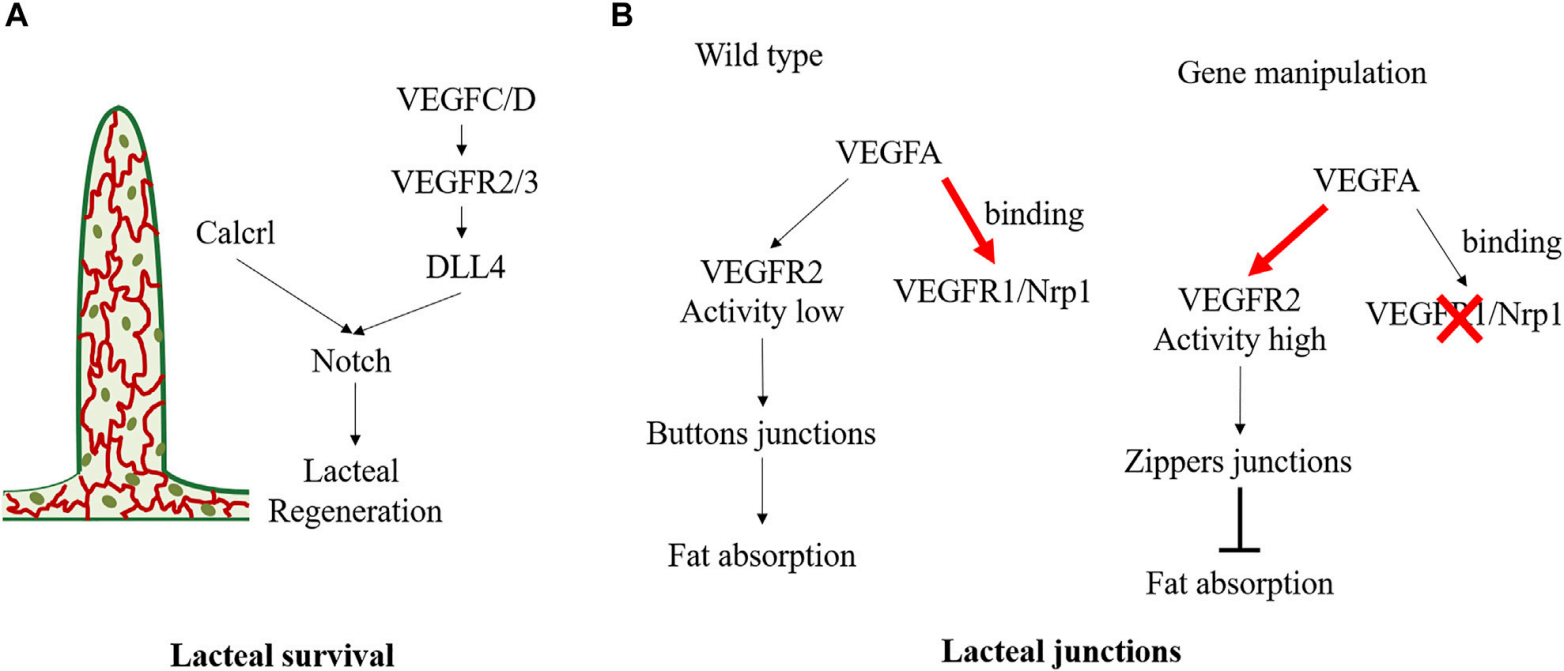
Molecular mechanisms regulating the status of lacteal survival and lacteal junctions. **(A)** VEGFC/D signals activate VEGFR2/3 and further promote lacteal DLL4 expression and Notch signaling. And Calcrl is also the upstream of Notch/DLL4 signaling in maintaining lacteal regeneration. **(B)** In physiological state, VEGFA binding to VEGFR1and Nrp1 on blood endothelial cells resulting in low VEGFR2 activity in lymphatic endothelial cell, which maintains the buttons junctions and facilitates fat absorption. Deletion of endothelial Nrp1 and VEGFR-1 results in high VEGFR2 signaling activity, leading to the formation of zippers junction and impairs fat absorption.

Lacteals are in permanent regeneration and slow proliferation to replace damaged cells (Norden and Kume, 2020). Defective growth of lymphatic vessels in the lacteals can lead to dietary lipid malabsorption and prevent obesity. Vascular endothelial growth factor (VEGF) receptor 3 (VEGFR3) is a vital mediator in lacteal survival (Figure 4A). *Chy* mice, with *Vegfr3* inactivated mutation, exhibited defective lymphatic vessels. *Chy* mice exhibited significantly increased TG accumulation in enterocytes and increased fecal lipid content, contributing to the reduced postprandial plasma TG levels and protecting against obesity. The mechanism is partly related to the mutant of *Vegfr3* significantly reducing nitric oxide production, which is required for CM to be released into circulation (Shew et al., 2018). Moreover, VEGF-C is an indispensable molecular regulator of intestinal lymph angiogenesis, although VEGF-D is also a ligand of VEGFR3 (Nurmi et al., 2015). Gut microbiota can influence VEGF-C production by villus macrophages and further affect the lacteal integrity. Because the recognition of microbial components *via* the toll-like receptors/myeloid differentiation factor 88 complex is a vital upstream signal for VEGF-C production, depletion of gut microbiota can break down this process, causing decreased VEGF-C expression and resulting in lacteal regression, specifically in jejunum and ileum (Suh et al., 2019). Genetical deletion of *Vegfc* (Vegfc^−/−^) can lead to lacteal regression without affecting other lymphatic beds, thereby influencing lipid absorption and enhancing excretion of FAs and cholesterol in faces. Vegfc^−/−^ mice exhibited improved glucose metabolism and were protected from HFD-induced obesity (Nurmi et al., 2015). Bernier-Latmani *et al.* found that Notch signaling also contributes to the continuous regeneration of lacteals (Bernier-Latmani et al., 2015). Notch ligand delta-like 4 (Dll4) is the ligand of the Notch receptor. Mice with genetic ablation of *Dll4* (Dll4^−/−^) in LECs exhibited shorter lacteals and the survival and migration of IEC were unable to maintain (Bernier-Latmani et al., 2015). VEGFR3 and VEGF2 activate Dll4 expression in lacteals. The altered VEGFC/D-VEGFR2/3 signaling may induce lipid absorption defects partly through Notch signaling. Moreover, Calcitonin receptor-like receptor (Calcrl) has also been reported as an essential upstream regulator of the Notch pathway (Figure 4A). Compared with control mice, mice with temporal and spatial deletion of *Calcrl* (Calcrl^fl/fL^/Prox1-CreER^T2^) developed intestinal lymphangiectasia and lacteals uptake lipids defecits and exhibited reduced weight gain (Davis et al., 2017).

#### 5.7.2 Lacteal junctions

There are button junctions and zipper junctions between endothelial cells in lacteal. The button junction has open and closed regions, which allows CMs transport, but the zipper junction only has the closed region, preventing CM uptake (Figure 4B). VEGF-A-VEGFR2 interaction can regulate endothelial cell junction, influence CMs production, and exert an obesity protection effect. Normally, VEGF-A binds VEGFR1 and Neuropilin1 (Nrp1) on blood endothelial cells. However, in *Nrp1*/*Vegfr1* deletion mice, the bioavailability of VEGF-A significantly increased, and signaling through VEGFR2 leads to lacteal junction zippering and CM uptake defection (Zhang et al., 2018). Compared with HFD wild-type mice, *Nrp1*/*Vegfr1* deletion mice showed decreased fat mass, improved glucose tolerance, and reduced plasma TG, total cholesterol, and HDL concentrations, and no hepatic steatosis (Zhang et al., 2018). Lymphatic *Dll4* mutation also contributes to a shift of button junction to continuous zipper junction, resulting in inefficient CM uptake. Moreover, Dll4^−/−^ mice exhibited a significantly blunted increase in plasma TG and free FA (Bernier-Latmani et al., 2015). In contrast, dexamethasone, a drug that induces the transition of the tracheal lymphatic zipper to button-like junctions, can reduce the number of zipper connections and increase CM uptake (Zhang et al., 2018).

It is worth noting that lymphatics are vital in maintaining intestinal homeostasis, impaired lacteals exacerbate intestinal mucosal inflammation and contribute to increased risk of obesity and cardiovascular disease (Jiang et al., 2019; Cifarelli et al., 2021). Mice heterozygous for Prospero-related homeobox1(Prox1^+/−^) had defective lymphatic vascular integrity, and the accumulation of lymph can lead to adipocyte proliferation and development of spontaneous adult-onset obesity (Harvey et al., 2005). In addition, CD36 is not only expressed in enterocytes but also highly expressed in lacteals. Mice with inducible *Cd36* deletion in LECs had increased discontinuous button junctions in lacteals and submucosal lymphatic vessels, resulting in lymph leakage (Cifarelli et al., 2021). Immune cells from lymph can traffic to adipose tissue causing proinflammatory changes, which are closely linked to systemic glucose intolerance. FAs from the lymph can expand mesenteric and visceral adipose depots, similar to previously mentioned Prox1^+/−^ mice (Cifarelli et al., 2021). In addition, lymphatic vessels also contribute to maintaining gastrointestinal homeostasis. Davis *et al.* reported that Calcrl^fl/fl^/Prox1-CreER^T2^ mice with lymphatic insufficiency impaired the resolution of intestinal inflammation from drug-induced acute mucosal injury and exhibited dilated lacteals and protein-losing enteropathy (Davis et al., 2017).

## 6 Conclusion and perspectives

Obesity poses a severe threat to human health. The disorder of dietary TG absorption dramatically affects the occurrence and development of metabolic diseases. Prolonged HFD feeding can change intestinal morphology, induce altered morphometric parameters and gastrointestinal motility, and impair the intestinal mechanical and immune barrier. In contrast, restricting TG absorption ameliorates the obesity phenotype, indicating a promising strategy for anti-obesity.

Dietary TG absorption involves various regulatory genes and proteins that play a vital role in regulating the related processes. Besides, the length of the carbon chains, the number of unsaturated bonds, and the location of FAs in dietary TG affect absorption efficiency and metabolic phenotypes. The undergoing studies are limited in: i) the expected efficacy is unsatisfied by intervening in certain essential genes or proteins (e.g., CD36, FABPs, DGAT, SAR1A, *etc.*); ii) lacking clinical safety and efficacy of the dietary TG absorption-related synthetic small biomolecules; iii) unexpected side effects by inhibiting certain key targets, such as severe gastrointestinal reactions (e.g., CD36, DGAT1 *etc.*), systemic dyslipidemia (CD36, caveolin-1, *etc.*), and other adverse reactions that interrupt the long-term treatment. Furthermore, differences in sex, living environment, circadian rhythms, and dietary nutrient structure also hunt the therapeutic benefits. Therefore, regulating dietary TG absorption to control HFD-induced obesity is still challenging.

We note that recently published high-quality studies have indicated some key targets of the intestinal lipid absorption process are promising in therapeutic prospects, such as MGAT2 inhibitors and intestinal-specific MTP inhibitors. TG absorption is an efficient and delicate process. Further research is needed to balance the metabolic benefits and side effects. Combination therapy that regulates multiple targets of intestinal lipid absorption may be an alternative and safe therapeutic approach.
